# A visual atlas of meiotic protein dynamics in living fission yeast

**DOI:** 10.1098/rsob.200357

**Published:** 2021-02-24

**Authors:** Wilber Escorcia, Vishnu P. Tripathi, Ji-Ping Yuan, Susan L. Forsburg

**Affiliations:** ^1^ Molecular and Computational Biology Program, University of Southern California, Los Angeles, CA 90089, USA; ^2^ Leonard Davis School of Gerontology, University of Southern California, Los Angeles, CA 45207, USA

**Keywords:** meiosis, histone, cohesin, microtubule, spindle assembly checkpoints, PCNA

## Abstract

Meiosis is a carefully choreographed dynamic process that re-purposes proteins from somatic/vegetative cell division, as well as meiosis-specific factors, to carry out the differentiation and recombination pathway common to sexually reproducing eukaryotes. Studies of individual proteins from a variety of different experimental protocols can make it difficult to compare details between them. Using a consistent protocol in otherwise wild-type fission yeast cells, this report provides an atlas of dynamic protein behaviour of representative proteins at different stages during normal zygotic meiosis in fission yeast. This establishes common landmarks to facilitate comparison of different proteins and shows that initiation of S phase likely occurs prior to nuclear fusion/karyogamy.

## Graphical abstract

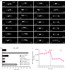

## 1. Introduction

Meiosis is a conserved cell differentiation pathway found in sexually reproducing eukaryotes. It is characterized by one round of DNA synthesis, followed by two nuclear divisions, resulting in haploid gametes (reviewed in [1–4]). In most organisms, the divisions are preceded by programmed double-strand breaks (DSB) followed by homologous recombination (HR) that facilitates separation of the homologous chromosomes during reductional meiosis I (MI) division (reviewed in [5]). The equational meiosis II (MII) division separates sister chromatids and more closely resembles mitosis in somatic or vegetative cells but is unusual in that it does not follow a period of DNA synthesis (reviewed in [4]).

A variety of meiosis-specific genes are recruited to facilitate these specialized events. These include additional recombination proteins, components of the synaptonemal complex or linear elements between chromosome homologues, proteins that contribute to kinetochore mono-orientation and meiosis-only cohesin proteins that link sister chromatids together. These specialists modify the basic architecture of cell division particularly to facilitate the meiosis-specific chromosome recombination and the MI division mechanisms [6–8]. The yeast *Schizosaccharomyces pombe* offers an excellent model system to study the process of meiosis. Normally haploid cells can be induced to conjugate with cells of the opposite mating type in conditions of nutrient limitation, forming transient zygotes. The initial landmark event following karyogamy is the formation of a telomere-led chromosome bouquet, which migrates rapidly back and forth in the length of the cell in a dynein-led movement called horse-tailing (HT) [9,10]. Meiotic S (meiS) phase and recombination occur during horse-tailing, which is followed by the MI and meiosis II (MII) divisions (reviewed in [11–13]). Genetic tools have been deployed to identify and characterize proteins that function throughout these different phases.

The advent of green fluorescent protein (GFP) and related colourful fluorescent tags has allowed the analysis of protein dynamics in living cells and these have been widely used in studies of fission yeast meiosis. However, variation in protocols and conditions make it challenging to compare the dynamics of proteins in the various stages of meiosis between different publications. In particular, many studies employ a temperature sensitive *pat1* mutant to drive a synchronous meiosis, even from haploids, but *pat1* disrupts some aspects of meiosis (e.g. [14–16]). In this report, we examine protein dynamics in meiosis using consistent strains, growth conditions and a standardized imaging protocol [17,18]. This allows us to generate an atlas of meiotic protein behaviour that will be a useful reference to many investigators studying this dynamic process.

## 2. Results and discussion

*Rationale*. We used a uniform growth strategy to induce mating and meiosis between cells of opposite mating types, followed by imaging of cells on agar pads (see Material and methods [17,19]). Importantly, aside from the tagged genes, the strains are normal haploids that were induced to mate, followed directly by zygotic meiosis. Reasonable synchrony was achieved solely by using appropriate growth conditions, and we did not employ any mutations such as *pat1-ts*, which can induce meiotic abnormalities (e.g. [14,16]). Thus, our strategy recapitulates as much as possible a ‘normal’ fission yeast meiosis.

### 2.1. Nuclear dynamics establish a reference

Hht1 and Hhf1 are the H3–H4 histone pairs that package DNA in fission yeast [20,21]. Because of its role in nuclear organization, Hht1 is used with fluorescent tags to mark the nuclear mass during mitotic and meiotic processes [19,22,23]. To establish an initial reference for meiotic progression, we employed cells carrying homozygous Hht1-mRFP and followed meiotic progression using live-cell imaging. We began our observations prior to karyogamy, during pre-fusion (PF), and followed through karyogamy/nuclear fusion (FS), horse-tailing (HT), metaphase I (MTI), MI and all the way up-to MII.

**Figure 1.**
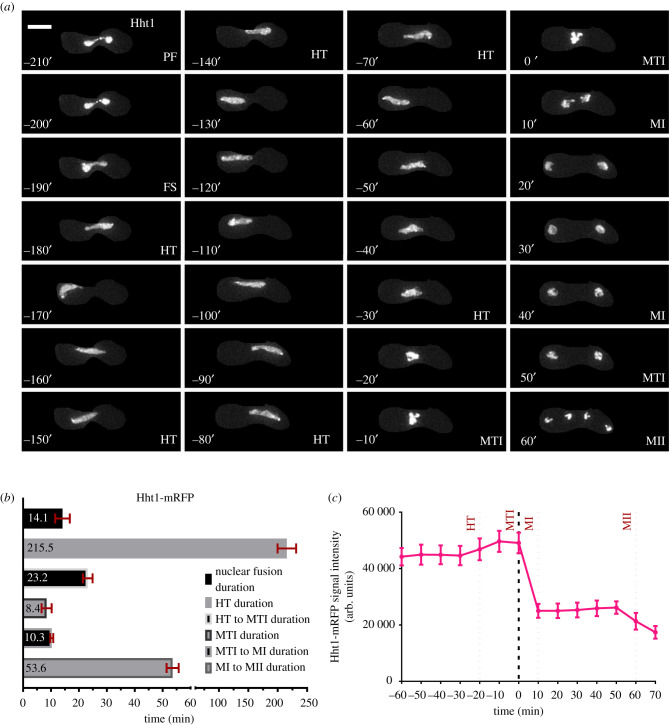
Nuclear dynamics of Hht1 defines signposts for meiotic events. (*a*) Live-cell image of a heterothallic unperturbed zygotic meiotic cell carrying fluorescently tagged histone 3 (Hht1-mRFP) (FY5608 × FY5609). Time lapse images were captured every 10 min for 8 h and selected frames are shown. More than 35 cells from at least two independent biological replicates movies were analysed and representative images presented. The panel shows hht1-mRFP dynamics during meiotic cycle encompassing different meiotic signposts as PF (PF: −210′), FS (FS: −190′), HT (HT: −180′ to −30′), MTI (MTI: −10′ to −0′), MI (MI: 10′–40′), MTII (MTII: 50′) and MII (MII: 60′). Numbers represent timing in minutes and the scale bars are equal to 5 µm. Metaphase I, just prior to MI, is considered as ‘t0″ and any events occurring prior to that are represented by negative timing numbers, while post metaphase I events are marked by positive numbers. (*b*) The durations of Hht1-mRFP dynamics in different meiotic events. (*c*) Quantitation of Hht1-mRFP fluorescence intensity. Average (mean) values of timing and fluorescence intensities of individual events, captured in more than 35 cells, randomly selected, from two independent biological samples are presented in (*b*) and (*c*) respectively. Error bars represent 95% class intervals.

To set a common landmark for timing comparisons, we used the clearly recognizable characteristics of MTI as a reference time = 0 (t0) minute (MTI: 0′). Events occurring prior to MTI are represented by negative timing numbers (e.g. −10′, −20′ etc.) and events occurring post MTI are indicated by positive numbers (e.g. 10′, 20′ etc.). Moreover, the timing numbers with landmarks (e.g. figure 1*a*: PF: −210′, FS: −190′) indicate the actual timing of the event shown in a particular image presented for data representation purposes. The durations of different events (e.g. figure 1*b*) are based on average timing of more than 35 cells, randomly selected, from two independent biological replicates.

In our initial examination of histone dynamics, we observed a bright, pan-nuclear Hht1-mRFP signal at prefusion (PF) (PF: −210′) with no signs of vigorous nuclear movements (figure 1*a*; electronic supplementary material, figure S1A). At karyogamy/nuclear fusion (FS), we observed a characteristic nuclear bridge structure, mixing of parental nuclei and doubling of nucleus size in the zygote relative to pre-fusion cells (FS: −190′). The period of nuclear fusion lasts for 14 min (mean timing = 14.1′ ± 2.6′) before the nuclear mass undergoes repeated oscillations for approximately 215 min (215.5′ ± 15.8′), representing horse-tailing (figure 1*b*). In this instance, the durations of the fusion (14 min) or horse-tailing stages (215 min) are the averages of timing durations of more than 35 cells, selected randomly from two independent biological replicates. When horse-tailing concludes, vigorous oscillation ceases, and the nuclear size starts to contract, with moderate to low nuclear movement (HT to MTI: −30′ to −10′) (figure 1*a*; electronic supplementary material, figure S1B-C). This transition phase of nuclear reconfiguration lasts for approximately 20 min (23.2′ ± 1.7′) followed by 8 min (8.4′ ± 1.8′) of MTI, where maximal nuclear compaction occurs and Hht1-mRFP signal intensifies (MTI: −10′ to 0′) (figure 1*a–c*).

To better represent the changes in hht1-mRFP signal intensities along with nuclear dynamics, we used the ‘Thermal’ look-up tables (LUT) feature of FIJI [24]. In this presentation, the yellow and red colour signals of hht1-mRFP represent higher intensities owing to higher compaction of the nucleus (electronic supplementary material, figure S1A). The nucleus in MI splits into two nuclear masses of roughly equal size (MI: 10′) (figure 1*a*; electronic supplementary material, figure S1B) that contain segregated sets of homologous chromosomes [25–27]. The transition of MTI to MI lasts for approximately 10 min (10.3′ ± 0.5′), whereas the average duration of MI to MII is approximately 50 min (53.2′ ± 2.2′) (figure 1*a,b*). During MTII, homologs continue to move toward opposite ends of the zygote. Final separation of the two nuclear masses into four daughter nuclei marks the completion of MII (MII: 60′) (figure 1*a*). At this stage, sister chromatid sets completely segregate and are readied for packaging into spores in the gamete-maturation phase that follows MII [25–27].

Our observations of H3 histone (Hht1-mRFP) dynamics generally agree with those of other live-cell studies [19,23,28–32]. The timing from nuclear fusion to MII takes approximately 4½ hours, which is in line with previous reports [23,28,33]. These histone dynamics established a baseline that we used to compare different strains and markers.

We observed that predictable changes in nuclear mass correlate with changes that define functional shifts in different markers of meiotic progression. As expected, there is a correlation between signal intensity changes and changes in nuclear size. Significant increase in Hht1-mRFP fluorescence intensity is observed during fusion and in each of the two metaphase steps preceding the meiotic divisions. This link is consistent with reports showing elevated histone content during DNA synthesis and increased nuclear condensation in metaphase [20,21,34–36].

### 2.2. S phase markers PCNA and Tos4

PCNA (SpPcn1) plays major roles in DNA replication, repair and translesion synthesis (revived in [37]). As a processivity factor, it interacts with both DNA polymerases δ and ε ([38]; reviewed in [39]). To study the nuclear dynamics and timings of meiotic S phase, we used a heterozygous cross between cells containing enhanced green fluorescence protein (EGFP)-Pcn1 [40] and hht1-mRFP. For the S phase markers, instead of metaphase I, we used the initial nuclear fusion stage (FS) to represent the t0 minute (FS: 0′), to resolve earlier events. Events prior to karyogamy or prefusion have negative timing (PF: −10′, −20′) and post fusion stages such as horse-tailing stage have positive timing numbers (HT: 10′, 20′ etc.)

**Figure 2.**
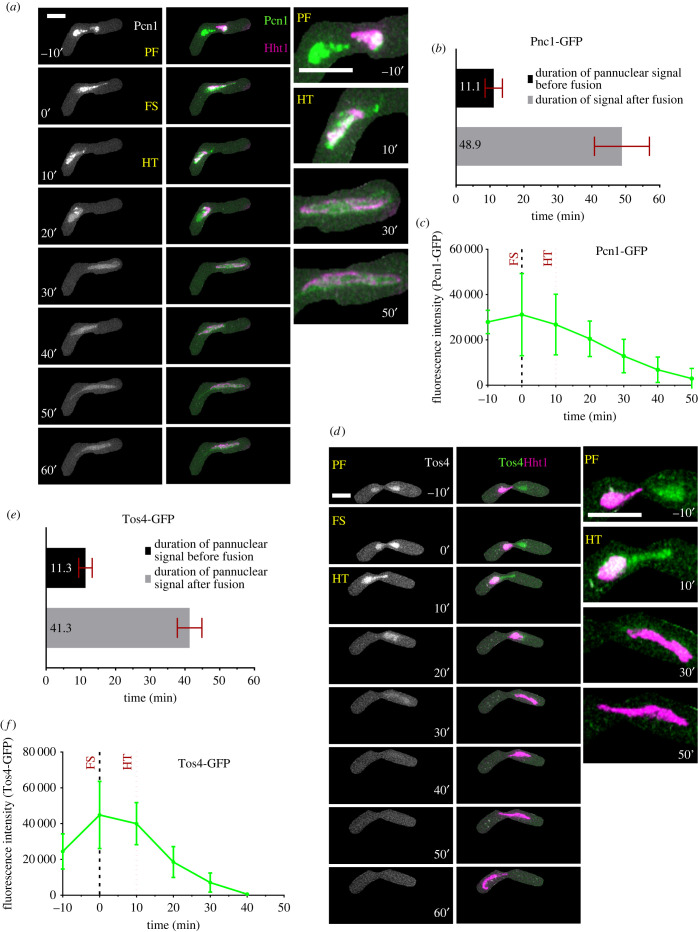
Nuclear dynamics of Pcn1 and Tos4 during meiotic S phase. (*a*) Live-cell images of zygotic meiotic cell carrying EGFP-tagged PCNA (EGFP-Pcn1) and mRFP-tagged Hht1 (3050 × 5615). Time lapse images were captured every 10 min for 8 h and selected frames are shown. The panel shows dynamics of EGFP-Pcn1 (column 1) and EGFP-Pcn1, Hht1-mRFP merged (column 2) during early meiotic events encompassing pre-fusion (PF), karyogamy (FS) and early horse-tailing (HT) stage. Column 3 is showing closeups of selected time points from column 2. Exclusively for S phase markers, the ‘t0’ is changed from metaphase I (MTI) to fusion (FS) stage. Events prior to FS are represented by negative numbers and post fusion events are by positive timing numbers. Numbers represent timing in minutes and scale bars equal 5 µm. (*b*) Durations of EGFP-Pcn1 foci signal disappearance. (*c*) Quantitation of EGFP-Pcn1 fluorescence intensity. (*d*) Time lapse images of a zygotic meiotic cells carrying Tos4-GFP and Hht1-mRFP (8848 × 5615). Panel showing dynamics of Tos4-GFP (column 1), Tos4-GFP and hht1-mRFP merged (column 2) during early meiotic events encompassing pre-fusion (PF), karyogamy (FS) and early horse-tailing (HT) stage. Column 3 shows closeups of selected time points from column 2. Numbers represent timing in minutes and scale bars represent 5 µm. (*e*) Durations of Tos4-GFP signal disappearance. (*f*) Quantitation of Tos4-GFP fluorescence intensity. To estimate timing and signal intensity, *n* = 9 (PCNA) and *n* = 15 (Tos4) cells from at least two independent biological replicate movies were analysed. Data in (*b*), (*c*), (*e*) and (*f*) represent mean values. Error bars represent 95% class intervals.

During the PF stage (PF: −10′), we observed bright and discrete EGFP-Pcn1 foci, similar to those observed in vegetative S phase [40]. These were distributed across both nuclei and persisted for about 10 min (11.1 ± 1.1′) (figure 2*a*,*b*). Notably, the histone signal is observed in just one parent, further indicating that fusion has not occurred. During karyogamy (FS: 0′), the foci remained distributed across the nucleus and the signal intensity increased (figure 2*c*). As cells moved from karyogamy to the horse-tailing stage, EGFP-Pcn1 signal remained distributed across the nucleus (HT: 10′) but localized in one part, which may indicate late replication activity in certain parts, possibly the nucleolus (HT: 10′–30′) (figure 2*a*). We observed an average of about 49 min (48.9′ ± 8.1′) from karyogamy before the EGFP-Pcn1 signal was lost. Notably, horse-tailing continued for another 160–180 min but Pcn1 signal remained diffused, which we infer indicates the conclusion of S phase.

Previously, in a *pat1*- induced meiosis, it was reported that DNA replication occurs at the beginning of the horsetail stage [34]. However, a previous study using fluorescently tagged Pcn1 in homothallic *h90*, is consistent with our study using heterothallic *h^−^* and *h^+^*, suggesting early S phase begins prior to karyogamy [41]. We speculate that initiation of premeiotic S phase is likely coordinated by trans-acting transcription factors that form after cell fusion but prior to nuclear fusion (e.g. [42]).

We examined another S-phase marker, Tos4, which is a transcription factor that accumulates in the nucleus during early S-phase [43–46]. It contains a fork-head associated (FHA) domain and is regulated by the G1/S transcription factors SBF (Swi4-Swi6 cell cycle box binding factor) [47,48]. Here again we used a heterozygous cross, with one parent including fluorescently tagged Tos4 (Tos4-GFP) and the other Hht1 (Hht1-mRFP), to examine its behaviour in meiosis. Like EGFP-Pcn1, we observed bright foci distributed throughout the nucleus prior to nuclear fusion (PF: −10′), which lasted for about 11 min (11.3′ ± 1.9′) (figure 2*d*,*e*). As cells moved to karyogamy, the signal intensity increased and reached its maximum (FS: 0′) (figure 2*f*). The Tos4-GFP signal gradually disappears as cells spend about 25–30 min in horse-tailing (HT: 30′) (figure 2*d*,*f*). The duration of Tos4 signal from fusion to early horse-tailing is observed for about 40 min (41.3′ ± 3.5′) (figure 2*e*). The initial dynamics and timing of Tos4-GFP accumulation in the nucleus are similar to those of EGFP-Pcn1, but without late focus formation during the later phase of HT with Pcn1 (figure 2*a*: HT: 20′). This suggests that Tos4-GFP is an early meiotic S phase marker with similar dynamics to vegetative S phase [45].

The consistent nuclear dynamics and timing of the two meiotic S phase markers, Tos4 and Pcn1, indicate that the activation of meiotic DNA replication starts prior to nuclear fusion. Indeed, given the approximately 25 min maturation time of GFP [49], it is likely to initiate even earlier than our observation.

### 2.3. Cohesin and pairing proteins

**Figure 3.**
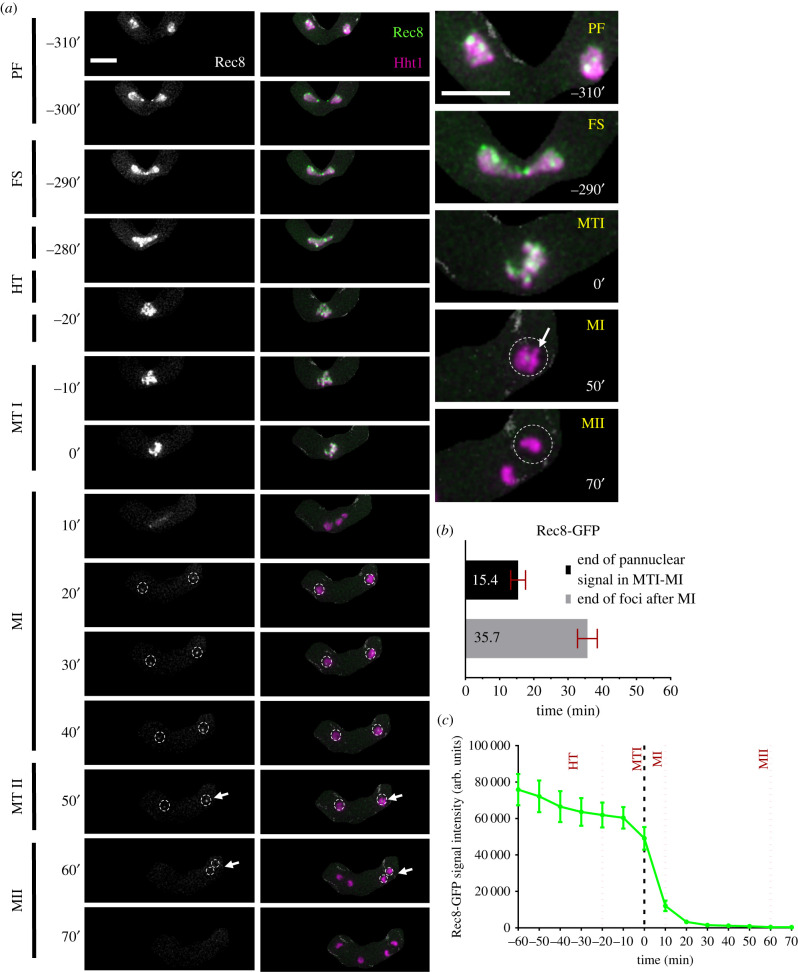
Rec8 cohesin dynamics. (*a*) Time-lapse images of meiotic cells carrying homozygous Rec8-GFP and Hht1-mRFP (7748 × 7840) were captured every 10 min for 8 h and selected frames are shown. The panel shows cohesin dynamics (column 1: Rec8-GFP) and (column 2: merged Rec8-GFP Hht1-mRFP) during different meiotic events covering pre-fusion (PF: −310′ to −300′), karyogamy (FS: −290′), horsetail (HT: −280′ to −20′), metaphase I (MTI: −10′ to 0′), MI (MI: 10′–40′), metaphase II (MTII: 50′) and MII (MII: 60′–70′). Column 3 shows closeup images of selected time frames from column 2. Numbers represent timing in minutes and scale bars represent 5 µm. (*b*) Durations of Rec8-GFP signal during metaphase I and MI. (*c*) Quantitation of Rec8-GFP fluorescence intensity. More than 35 cells from at least two independent biological replicates movies were analysed and representative images presented. Data presented in (*b*) and (*c*) represent mean timing and fluorescence intensities, respectively. Error bars represent 95% class intervals.

Rec 8 is the alpha-kleisin subunit of the meiotic cohesin complex in fission yeast [28,50–52]. Following meiotic DNA synthesis, it keeps sister chromatids cohered at the centromere until just before MII, when it is degraded to allow for sister chromatid separation [53–56]. We examined meiotic progression in strains in a homozygous cross with Rec8-GFP and Hht1-mRFP (figure 3).

During prefusion (PF: −310′ to −300′), we observed discrete and bright Rec8-GFP foci distributed across the nucleus (figure 3*a*). This pattern does not change during nuclear fusion (FS), horse-tailing (HT), and metaphase I (MTI) (FS to MTI: −290′ to 0′). From the beginning of horse-tailing until the end of metaphase I, Rec8 distribution, timing and movement tracks those of H3 histone. A drastic change in signal intensity and nuclear pattern occurs as cells move from metaphase I (MTI) to MI. Within approximately 15 min (15.4′ ± 2.2′) of MI, Rec8-GFP signal decreases until it becomes a single focus in each daughter nucleus (MTI to MI: 0′–20′), consistent with centromere localization [51] (figure 3*b*,*c*). This focus remains until metaphase II (MI to MTII: 20′–50′) and disappears just before cells enter meiosis II (MII: 60′–70′). The complete loss of Rec8-GFP signal, which precedes the final equational division of MII, takes about 35 min (35.7′ ± 2.9′) from meiosis I (MI: 20′–50′) (figure 3*b*).

Our results agree with previous observations that Rec8 is detected in cells that have not yet undergone karyogamy [51,52,57], which we observe are already in S phase. This is consistent with the nuclear organization function of Rec8 during recombination and metaphase I condensation [28,35,50]. We have shown previously that disruption of DNA replication dynamics changes the relative timing of Rec8 in these events [19,58–60], making this a particularly sensitive marker for meiotic progression.

**Figure 4.**
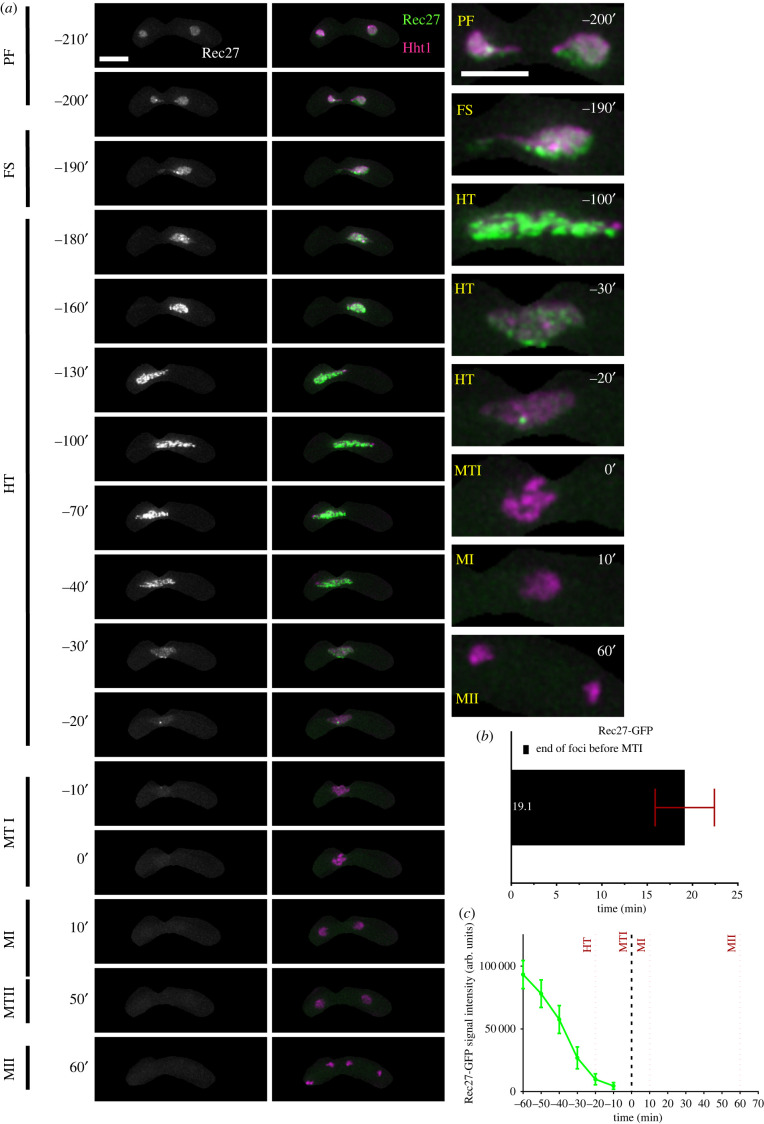
Nuclear dynamics of linear element protein, Rec27. (*a*) Time-lapse images of meiotic cells carrying Rec27-GFP and Hht1-mRFP (7777 × 7779) were captured every 10 min for 8 h and selected frames are shown. Column 1 (Rec27-GFP) and column 2 (merged Rec27-GFP Hht1-mRFP) show linear element dynamics during different meiotic events covering PF, FS, HT, MTI, MI, MTII and MII. Numbers represent timing in minutes and scale bars represent 5 µm. (*b*) Durations of Rec27 foci elimination before metaphase I. (*c*) Quantitation of Rec27-GFP fluorescence intensity. More than 35 cells from at least two independent biological replicates movies were analysed and representative images presented. Data in (*b*) and (*c*) represent mean timing and fluorescence intensities, respectively. Error bars represent 95% class intervals.

Rec27, a meiotic recombination protein, is a part of the linear element (LinE) proteins that bind along the axes of homologous chromosomes to regulate chromosome pairing, programmed double-strand breaks (DSB), and genetic recombination [61–64]. We used heterothallic strains containing homozygous Rec27-GFP and Hht1-mRFP and followed them from prefusion to MII (figure 4). In early events, prior to nuclear fusion (PF), we observed a diffused, pan-nuclear Rec27-GFP signal that later turned into discrete puncta (PF: −210′ to −200′) (figure 4*a*). As cells entered karyogamy, the number and signal intensity of these Rec27-GFP foci increased (FS: −190′). During horse-tailing, the size of Rec27-GFP foci increased and changed into long linear structures (HT: −180′ to −40′). As cells approached metaphase I, we observed gradual removal of those linear structures, converting into smaller foci with reduced signal intensity and number. This was followed by the complete elimination of Rec27-GFP signal (HT: −30′ to −0′). The disappearance of linear element signal happens about 20 min (19.1′ ± 3.2′) prior to the onset of metaphase I (figure 4*b*). We did not observe Rec27-GFP signal in metaphase I or following MI and MII (MTI to MII: −10′ to 60′) (figure 4*a*,*c*).

Similar to our observation with Rec8-GFP and S-phase specific markers, the linear element protein Rec27-GFP is first observed in pre-fusion nuclei. Given our data with Pcn1 and Tos4, we infer that the Rec27 protein accumulates in the nucleus during pre-meiotic S phase and it may have some direct or indirect role in DNA replication. This observation is consistent with gene expression data suggesting Rec27 is expressed before premeiotic S phase [65]. Previously, using nuclear spreads, it has been shown that Rec27 eventually forms distinct structures associated with linear elements [61]. We observe that Rec27 foci begin to disappear as cells transition from late horse-tailing into metaphase I. This observation reflects the end of Rec27 function in chromosome alignment and recombination [61–64,66]. Since disruption of Rec27 dynamics is associated with chromosome mis-segregation [19], the time-frame before nuclear condensation suggests a promising stage to study LinE proteins in the process of crossing-over and chiasma resolution [35,67].

**Figure 5.**
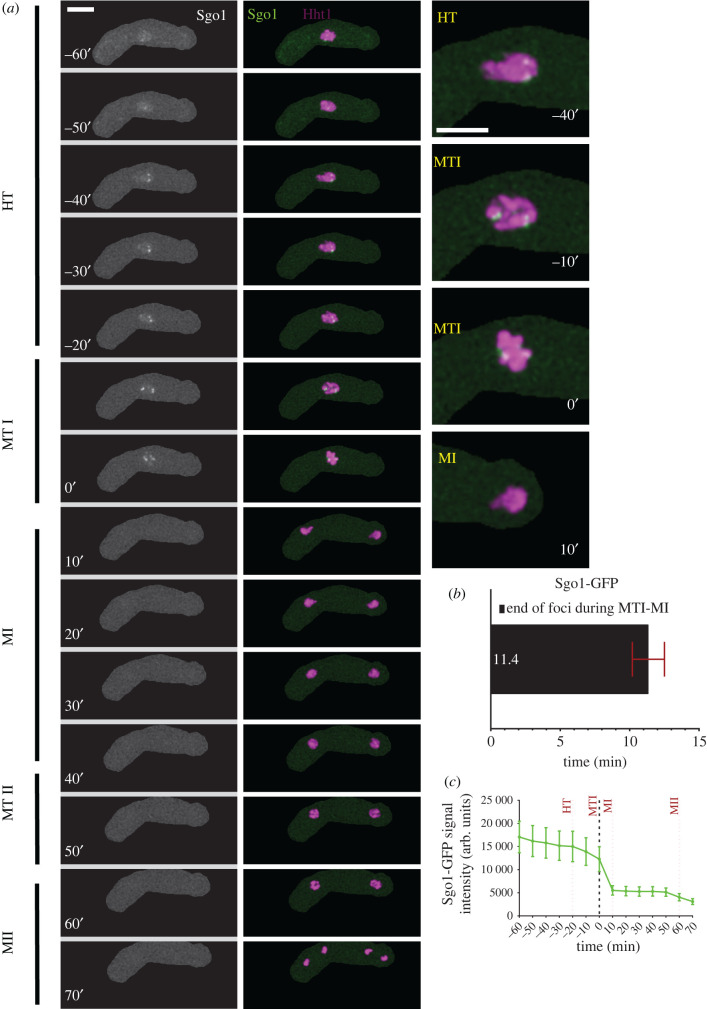
Nuclear dynamics of Shugoshin (Sgo1) protein. (*a*) Time-lapse images of meiotic cells carrying Sgo1-GFP and Hht1-mRFP (7864 × 7865) were captured every 10 min for 8 h and selected frames are shown. Panel show nuclear dynamics during different meiotic events covering HT to MII. Numbers represent timing in minutes and scale bars represent 5 µm. (*b*) Durations of Sgo1-GFP foci disappearance during metaphase I. (*c*) Quantitation of Rec27-GFP fluorescence intensity. More than 35 cells from at least two independent biological replicates movies were analysed and representative images presented. Data in (*b*) and (*c*) represent mean timing and fluorescence intensities, respectively. Error bars represent 95% class intervals.

The meiotic inner centromere protein, Shugoshin (Sgo1), along with phosphatase protein 2A (PP2A), helps to protect Rec8 from proteolytic degradation at the centromere prior to MI [53,54,56]. We observed meiosis in cells carrying homozygous alleles of Sgo1-GFP and Hht1-mRFP. Sgo1-GFP signal emerges when nuclear oscillation slows down in late horse-tailing. At this stage, Sgo1-GFP is diffused throughout the nucleus (pan-nuclear) but then resolves into discrete foci. These foci and nuclear signal remain visible for another 50–55 min before disappearing during the transition between metaphase I to MI (HT to MTI: −60′ to 0′) (figure 5*a*). These punctate foci disappear within 11 min (11.4′ ± 1.1′) of the metaphase I to MI transition (MTI to MI: 0′–10′) (figure 5*b*). We did not observe any Sgo1 foci or fluorescence signal after cells transitioned into MI and could only capture background fluorescence (MTI to MII: 0′–70′) (figure 5*a*,*c*). The emergence of Sgo1-GFP foci approximately an hour before metaphase I is consistent with its role as protector of centromeric Rec8 degradation prior to meiosis I [53–55]. Our data agree with other studies showing that Rec8-GFP is removed from chromosome arms but not the centromere, where Sgo1 localization prevents cleavage [53,60].

**Figure 6.**
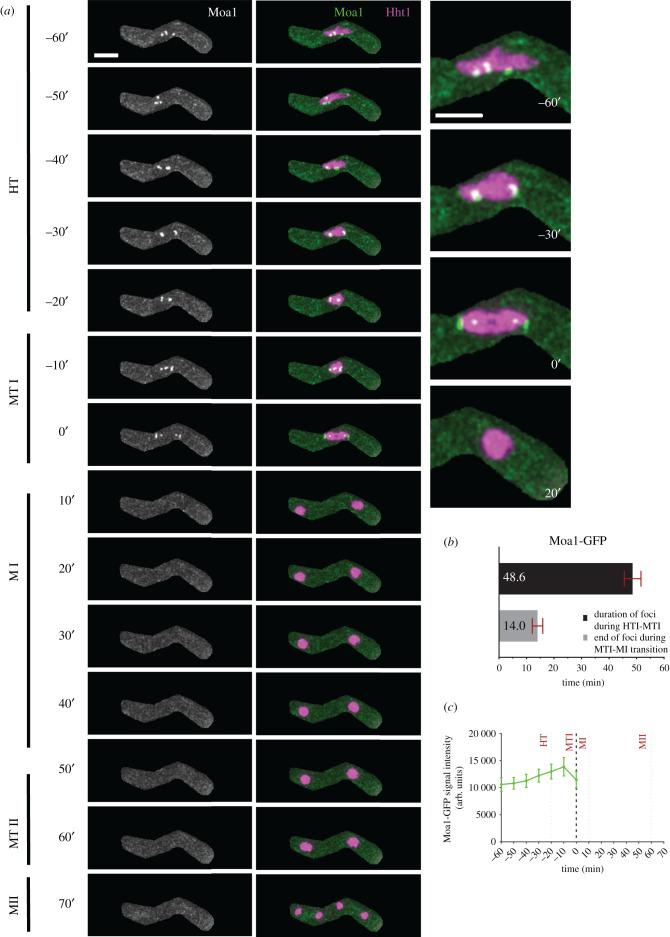
Nuclear dynamics of a kinetochore regulator Moa1. (*a*) Time-lapse images of meiotic cells carrying Moa1-GFP and Hht1-mRFP (8047 × 8048) were captured every 10 min for 8 h and selected frames are shown. Panel shows nuclear dynamics during different meiotic events covering HT to MII. Numbers represent timing in minutes and scale bars represent 5 µm. (*b*) Durations of Moa1-GFP foci during horsetails and metaphase I. (*c*) Quantitation of Moa1-GFP fluorescence intensity. More than 35 cells from at least two independent biological replicates movies were analysed and representative images presented. Data in (*b*) and (*c*) represent mean timing and fluorescence intensities respectively. Error bars represent 95% class intervals.

Moa1 (monopolin), a meiosis-specific kinetochore protein, is required to establish monopolar attachment of sister chromatids during MI [27]. It associates with Rec8 at the centromere and helps to stabilize Sgo1, thereby ensuring protection of centromeric cohesion, which is crucial for separation of homologous chromosomes [26,27,68,69]. Similar to Sgo1-GFP, we followed zygotic meiosis in cells carrying homozygous alleles of Moa1-GFP and Hht1-mRFP (figure 6). We did not see any GFP signal in early stages of PF, FS or early HT (data not shown). However, we saw discrete Moa1-GFP foci appear during later horse-tailing (HT: −60′) (figure 6*a*). Moa1-GFP foci remain visible for about 48 min (48.6 ± 3′) during late horse-tailing and early metaphase I (HT to MTI: −60′ to 0′) (figure 6*a*–*c*). The foci are initially dispersed around the nucleus and then cluster together at discrete spots at the poles of the condensing nuclei (MTI: −10′ to 0′). During metaphase I (MTI: 0′), Moa1-GFP forms a bright structure at the poles of the separating nuclei, presumably at the kinetochore, with a less intense dot just inside the nuclear mass. Within 14 min (14′ ± 2′) of the metaphase I to MI transition (MTI to MI: 0′–10′), we observe a complete loss of Moa1-GFP foci. We did not see any signal post anaphase I (MI to MII: 10′–70′). Moa1 is reported to localize in the central core of the centromere, where it interacts with Rec8, ensuring that sister chromatids face the same orientation prior to homolog separation [27,70,71]. The role of Moa1 in mono-orientation is evident from the separation of foci clusters that occurs as cells approach metaphase I. This signal pattern is similar to that of Sgo1-GFP, thereby lending additional support to their functional association [54,55].

### 2.4. Recombination and repair

**Figure 7.**
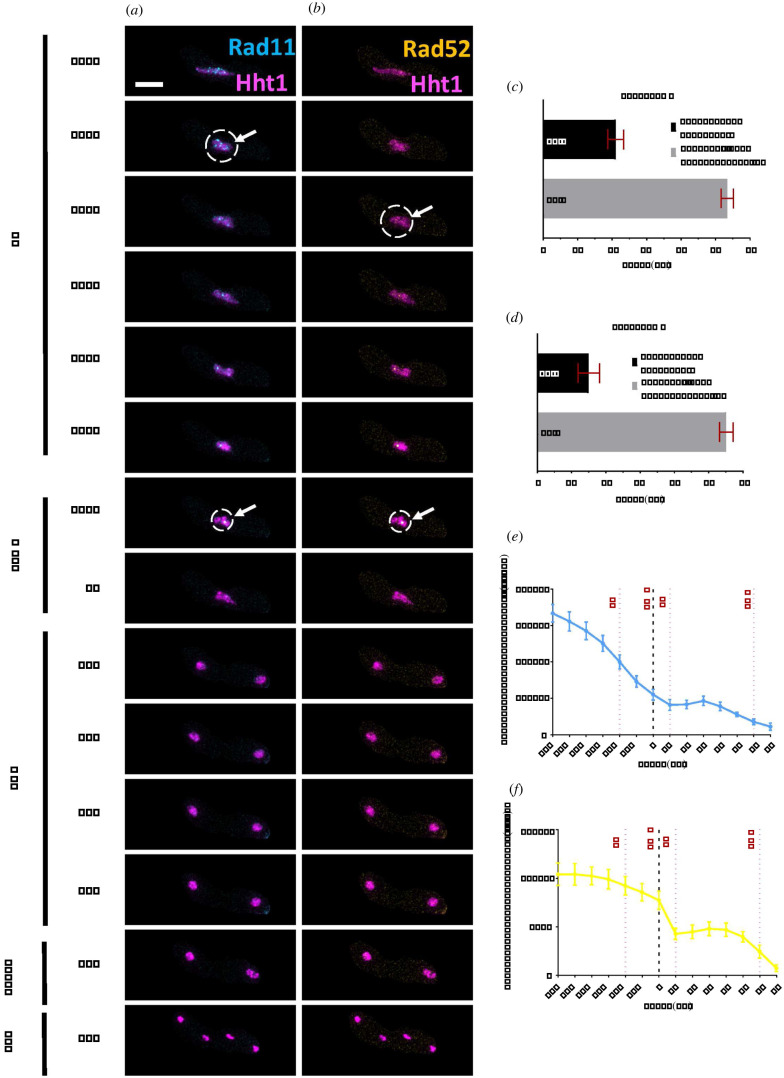
Nuclear dynamics of Rad11 (RPA) and Rad52 proteins. (*a*) Time-lapse images of meiotic cells carrying Rad11-CFP, Rad52-YFP and Hht1-mRFP (7327 × 7328) were captured every 10 min for 8 h and selected frames are shown. Panels (*a*) merged Rad11-CFP, Hht1-mRFP and (*b*) merged Rad52-YFP, Hht1-mRFP, show nuclear dynamics during different meiotic events encompassing HT to MII. Numbers represent timing in minutes and scale bars represent 5 µm. White arrows and circles show the presence of Rad11 and Rad52 foci signals. More than 35 cells from at least two independent biological replicates movies were analysed and representative image presented. (*c*,*d*) Durations of Rad11 (*c*) and Rad52 (*d*) foci signal loss, prior to metaphase I stage. (*e*,*f*) Quantitation of Rad11-CFP (*e*) and Rad52-YFP (*f*) fluorescence intensity. More than 35 cells from at least two independent biological replicates movies were analysed and representative images presented. Data in (*c*–*d* and *e*–*f*) represent mean values of timing and fluorescence intensities, respectively. Error bars represent 95% class intervals.

Rad11 (Ssb1) is the fission yeast orthologue of replication protein A (RPA), which binds to single stranded DNA (ssDNA) resulting from replisome progression in DNA synthesis and processing of DSB during DNA repair and homologous recombination [19,59,72,73]. To observe signal dynamics of recombination and repair proteins, prior to and during the meiotic nuclear divisions, we examined cells with fluorescently marked RPA (Rad11-CFP), Rad52 (Rad52-YFP) and Hht1(Hht1-mRFP). During prefusion (PF: −270′), we observed a pan-nuclear Rad11-CFP signal, which later forms discrete foci distributed across the nucleus from karyogamy (FS) until late horse-tailing (HT) stages, when the nuclear oscillation starts slowing down (PF to HT: −270′ to −50′ (electronic supplementary material, figure S2A)). As cells progressed towards the end of the horse-tailing, the number of foci and overall fluorescent intensity decreased and was finally lost before metaphase I (HT to MTI: −70′ to 0′), (figure 7*a*,*e*). The average mean time of the disappearance of these discrete foci, prior to the metaphase I, is about 20 min (21′ ± 2.3′) (figure 7*c*). From metaphase I onwards, we did not observe any foci. After MI, and for the next 53 min (53.4′ ± 1.8′), Rad11-CFP signal dissipate in the newly formed nuclei and finally ended before MII (MI to MII: 0′–70′) (figure 7*c*,*e*).

Rad52, a DNA recombination protein, acts with RPA and Rad51 to regulate recombination, which facilitates reductional division of the homologous chromosomes in meiosis I [74–77]. In our analysis, we did not see any Rad52-YFP signal during the early stages (PF to HT: −270′ to −50′) including prefusion, karyogamy, or early horse-tailing (electronic supplementary material, figure S2*b*). We noticed the emergence of Rad52 signal during late horse-tailing, the time when cells are undergoing recombination and repair events. We observed discrete punctate foci during late horse-tailing. These decrease in intensity and diffuse as the cells enter metaphase I (HT to MTI: −50′ to 0′) (figure 7*b*). Rad52 foci lasts about 15 min (15′ ± 3′) prior to metaphase I, which is 5 min later than Rad11 (figure 7*d*). We also noticed that, prior to and during metaphase I, some of the Rad11-CFP foci colocalized with Rad52-YFP foci (HT to MTI: −50′ to −10′) (figure 7*a*,*b*). Both Rad52 and RPA puncta were resolved by the time cells proceeded through the MI division. We observed a similar, diffuse nuclear Rad52 signal for about 55 min (55′ ± 1′) after MI until it finally disappeared (figure 7*d*,*f*).

These results indicate that proteins involved in replication and repair span the timing from pre-fusion to metaphase I. Rad11, a large subunit of RPA, is associated with DNA synthesis, repair and recombination [72]. The presence of discrete Rad11-CFP foci across the nucleus during prefusion resembles the nuclear dynamics of Pcn1, Tos4 and Rec8. This result is consistent with premeiotic S-phase initiating during the prefusion stage. The nuclear dynamics of Rad11 during late horse-tailing, where CFP foci start to decrease in number and intensity resembles those of Rec27, suggest the timing of recombination resolution.

Rad52 is a recombination mediator that either promotes Rad51 or antagonizes Dmc1 binding to RPA-coated presynaptic filaments during recombination [74–76]. Which recombinase follows Rad52 association with RPA largely determines recombination dynamics in meiotic prophase [74]. Our observations show that Rad52-YFP signal is diffused until late horse-tailing, when it forms punctate foci. Timing of Rad52 foci emergence and its colocalization with Rad11 foci during late horse-tailing suggest the timing of recombination and repair activities, which finally concludes before metaphase I, when both signals disappear.

### 2.5. Spindle and spindle checkpoint dynamics

**Figure 8.**
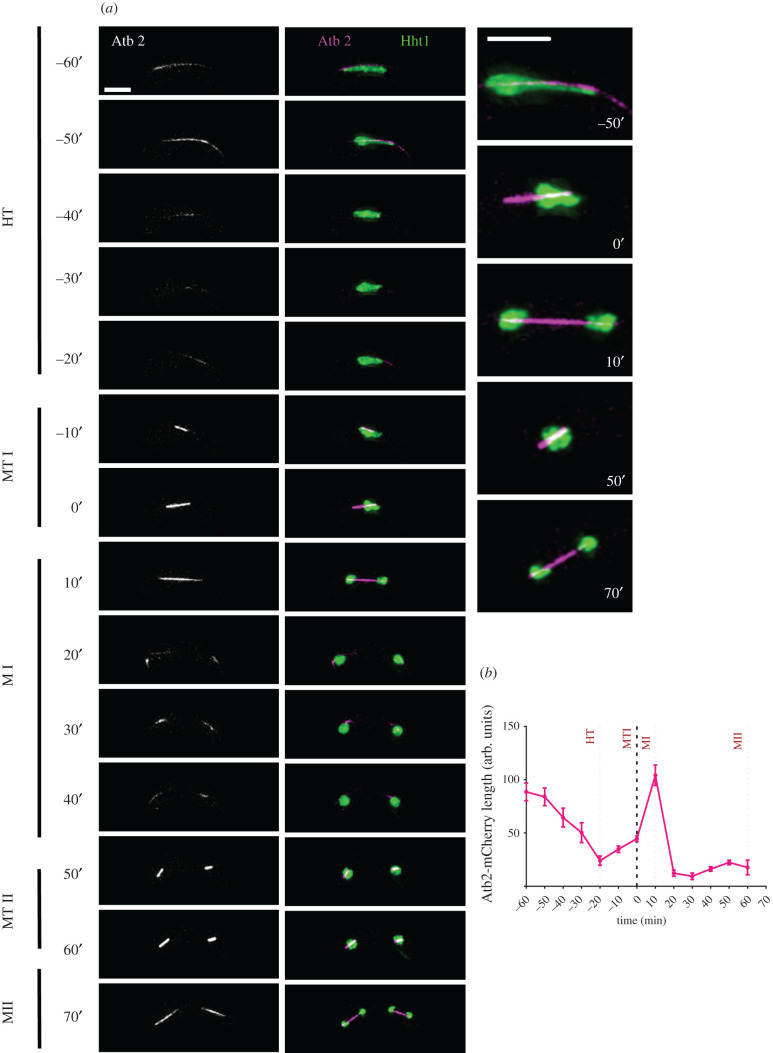
Meiotic spindle (Atb2-mCherry) dynamics. (*a*) Time-lapse images of unperturbed zygotic meiotic cells carrying fluorescently tagged heterozygous histone (Hht1-GFP) and tubulin (Atb2-mCherry) (8055 × 8203) were captured every 10 min for 8 h and selected frames are shown. Panel show spindle dynamics covering different meiotic events from HT to MII. Column 3 are a closeup images of selected time frames from middle panel (merged Atb2-mCherry and Hht1-GFP). Numbers represent timing in minutes and scale bars equal 5 µm. More than 35 cells from at least two independent biological replicates movies were analysed and representative image presented. (*b*) Quantitation of the length of microtubule. Data represent average length of microtubules. Error bars represent 95% class intervals.

The final group of proteins we studied are involved in microtubule dynamics and the spindle checkpoint. The spindle protein Atb2, encoding α-tubulin, is a constituent of cytoplasmic microtubule organization and is actively involved in establishment and maintenance of cell polarity and cell shape [78,79]. We crossed strains heterozygous for Atb2-mCherry [80] and Hht1-GFP, and imaged meiotic progression from pre-fusion to MII. Initially, during prefusion, we observed a bundle of microtubules (Atb2-mCherry) on one end of the cell and the Hht1-GFP signal on the other (PF: −220′ to −200′) (electronic supplementary material, figure S3A). As cells move to karyogamy, the bundle of microtubules approach the Hht1-GFP nucleus (FS: −200′ to −180′). Nuclear oscillations initiate with horse-tailing (HT: −160′). The microtubules show repeated expansion and contraction as they move across the cell (HT: −160′ to −40′; electronic supplementary material, figure S3, and HT: −60′ to −40′, respectively; figure 8*a*). At the end of horse-tailing microtubules briefly disappear, and the Atb2-mCherry signal returns as a spindle prior to metaphase I (HT: −50′ to −20′; figure 8*a*,*b*); this gradually increases in length and fluorescence signal intensity during the prophase I stage of MI (figure 8*a*,*b*; electronic supplementary material, figure S3B). At metaphase I, which lasts for about 12 min (12.3′ ± 1.5′), there is an increase in Atb2-mCherry signal intensity with slight change in spindle lengths (MTI: 0′) (figure 8*a*; electronic supplementary material, figure S3B). At anaphase I (MI), microtubules elongate outwards and reach to the cell's tip. The elongated microtubules carry newly separated daughter nuclei to the opposite ends of the cells and the process lasts for about 11 min (10.9′ ± 1′) (MI: 10′–20′) (figure 8*a*,*b*; electronic supplementary material, figure S3B). Next, for about 23 min, microtubules undergo depolymerization and reappearance of Atb2-mCherry signals showing completion of MI and the beginning of prophase II of MII stage (MI: 40′) (figure 8*a*,*b*; electronic supplementary material, figure S3B). Finally, at the end of MII the Atb2-mCherry signals disappear and four completely separated daughter nuclei form (MII: 70′). The duration of metaphase II is approximately 12 min (12.3′ ± 1.5′) while anaphase II lasts for about 14 min (14.6′ ± 1.8′) (electronic supplementary material, figure S3B). The spindle length and signal intensity in MI and MII are proportional to the respective nuclear mass (hht1-GFP) (figure 8*b*).

In *S. pombe* meiosis, microtubules play crucial roles from early stages including fusion and horse-tailing [81]. Our prefusion and karyogamy stage data are consistent with previous work showing a bundle of microtubules mobilizing and forming an X-shaped structure [9]. Data from MI and II stages are in alignment with previous reports showing three distinct steps in spindle elongation: prophase, metaphase characterized by constant spindle length with increased signal intensity, and anaphase, when microtubules elongate further leading to the edge of the cell with slight change in the signal intensities [71,82–84].

**Figure 9.**
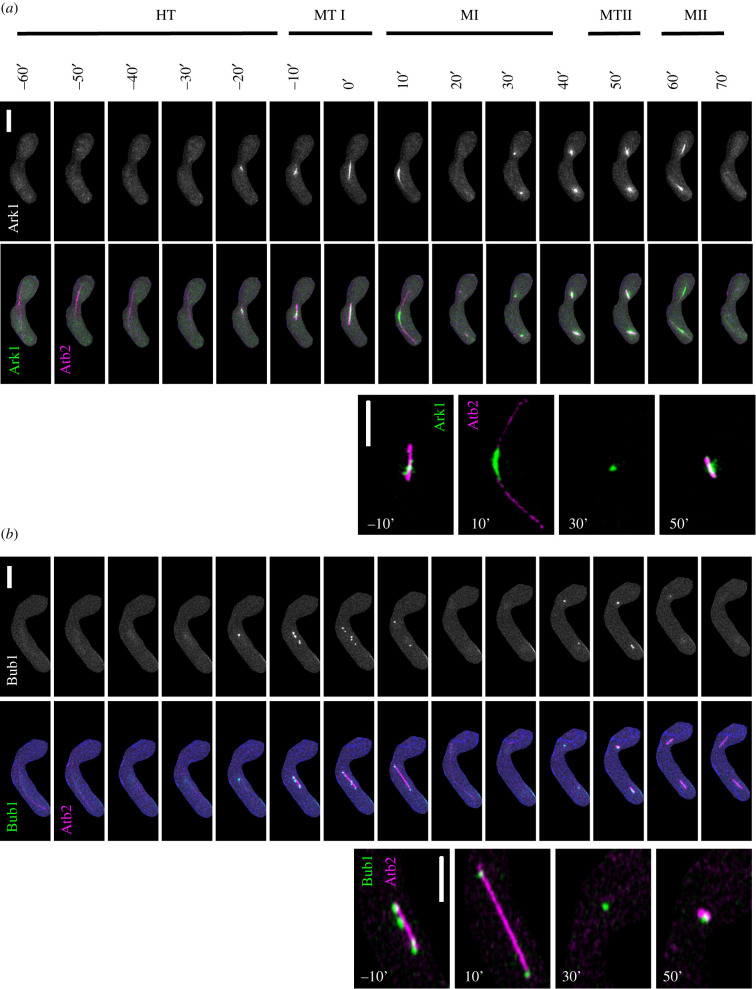
Nuclear dynamics of Aurora B (Ark1) and Bub1 kinase. Live-cell images of unperturbed zygotic meiotic cells carrying fluorescently tagged (*a*) Aurora B kinase (Ark1-GFP) and microtubule (Atb2-mCherry) (8053 × 8056) and (*b*) Bub1 kinase (Bub1-GFP) and microtubule (Atb2-mCherry) (8055 × 8067). Panels show nuclear dynamics during different meiotic events encompassing HT (HT: −60′) up to MII (MII: −70′). The 3rd and 6th rows are the images of selected time frames from the 2nd and 5th rows, respectively. Numbers represent timing in minutes and scale bars represent 5 µm. Representative images of more than 35 cells from at least two independent biological replicate movies are presented. Quantitative data presented in electronic supplementary material, figure S4.

Aurora-B kinase (*Sp*Ark1) is a serine/threonine kinase that ensures phosphorylation of substrates involved during chromosome condensation, activation of spindle assembly checkpoints and correction of erroneous kinetochore–microtubule attachments [25,83,85–87]. To examine its dynamics in zygotic meiosis with reference to microtubules, we used heterothallic strains containing Ark1-GFP and Atb2-mCherry. We did not see any Ark1-GFP signal during early events including prefusion, karyogamy or early horse-tailing stages (data not shown). The first Ark1-GFP signal was observed during the later stage of horse-tailing (HT: −20′), just prior to the beginning of metaphase I (figure 9*a*). Initially, for about 9 min (8.9′ ± 1.1′), it appeared as a discrete bright punctate focus, located in the central region of Atb2 (figure 9*a*; electronic supplementary material, figure S4A). At metaphase I, the number and fluorescence intensity of Ark1-GFP foci increased drastically and were distributed across the length of the spindle except on the two poles (MTI: −10′ to 0′) (figure 9*a*; electronic supplementary material, figure S4B). This scattered pattern of Ark1-GFP foci signal lasted for about 13 min (13.7′ ± 1.6′) (electronic supplementary material, figure S4A). At anaphase I (MI: 10′), Ark1-GFP signal moved towards the midzone and localized to the midbody of the extended spindles for about 14 min (14.3′ ± 2′). At this stage, the fluorescence signal intensity of Ark1-GFP reached a maximum (electronic supplementary material, figure S4B). After completing anaphase I, Ark1-GFP briefly disappeared for about 13 min (13.4′ ± 1.7′) before showing similar dynamics in MII: reappearance of Ark1-GFP signal during prophase II for 8 min (8.3′ ± 1.3′), redistribution to the spindles length during metaphase II for 17 min (17.7′± 1.5′) and back to the midbody on the spindles during anaphase II for 9 min (8.9′ ± 1.1′) following the similar pattern as observed in meiosis I (MI- MII: 30′–60′) (figure 9*a*; electronic supplementary material, figure S4A, B). Ark1-GFP signal disappeared after the completion of sister chromatid segregation at MII.

Our results showing appearance of discrete bright Ark1-GFP foci, near mid spindle region, agree with previous reports [25,83,87] and are consistent with enrichment on centromeres that might be aligned on the spindle. Sgo1 shows similar dynamics during prometaphase and metaphase and Ark1 colocalizes with Sgo1 at centromeres and spindle pole bodies (SPBs) [85,87]. The localization of Aurora kinase on peri-centromeres during prometaphase has been confirmed using ChIP [88]. The durations and dynamics of Ark1-GFP signals are in alignment with spindle polymerization, suggesting its crucial role in proper spindle maintenance. Our Ark1 dynamics of MII aligned with MI and mitotic cell cycles [25,83,87,89].

Next we examined an essential component of spindle assembly checkpoint, Bub1, a serine/threonine kinase, required for the protection of meiotic centromeric cohesion [90,91]. Previously, its dynamics were examined in *pat1* synchronized meiosis but how it behaves in an unperturbed meiosis was not tested [71,90]. Here, we used heterozygous cells carrying GFP-tagged Bub1 (Bub1-GFP) and mCherry-tagged Atb2 (Atb2-mCherry) and followed zygotic meiosis (figure 9*b*). We did not observe any Bub1-GFP signal until late horse-tailing when we saw a faint pan-nuclear signal (HT: −40′), which later formed a bright, discrete focus (HT: −20′). The pattern remained visible for about 18 min (18.3′ ± 2.3′) during late horse-tailing stage (electronic supplementary material, figure S4C). As cells reached metaphase I, the single Bub1-GFP focus split into a maximum of six foci distributed across the metaphase I spindle (figure 9*b*). Bub1-GFP signal intensity increased drastically and reached to the maximum, which lasted for about 11 min (11.4′ ± 1.2′) (MTI: 0′) (figure 9*b*; electronic supplementary material, figure S4C, D). At meiosis I, the signal across the spindle diffused and coalesced at a single focus on each daughter nucleus. Previous work suggests that Bub1-GFP enrichment at the ends of the spindle represents its localization at the kinetochores (MI: 10′) [71]. This stage lasts for 10 min (10.3′ ± 0.5′) (electronic supplementary material, figure S4C). As cells complete anaphase I, the Bub1-GFP signal disappears and returns after 20 min (20.9′ ± 1.7′) post anaphase I, when again it appears in puncta for about 10 min (10.3′ ± 0.6′) (figure 9*b*; electronic supplementary material, figure S4C). As cells enter metaphase II, the signal diffuses and distributes across the spindle in the same fashion as in metaphase I (MI-MII: 40′–60′). In this state, Bub1-GFP signals remain visible for 18 min (18.9′ ± 1.4′) before disappearing during anaphase II (electronic supplementary material, figure S8C). We did not see any Bub1-GFP signal during MII. Bub1 plays an important role in centromeric cohesion protection and proper Sgo1 localization to the pericentromeric regions [54,90,92]. Bub1 also plays a crucial role in Ark1 localization to the centromere regions [25,54,85]. Our results show that Bub1 and Ark1, during prophase and metaphase stages, show similar timing and behaviour. There is a difference during anaphase, when Ark1 leaves the centromeric region and localizes to the midzone of spindle, while Bub1 remains attached to the centromeres. Our result also shows different Bub1 dynamics during MI and MII, as it remains attached to the centromere in MI, but we could not see any signal during MII. This supports previous findings of Bub1's role in monopolar attachment [25,71].

## 3. Concluding remarks

**Figure 10.**
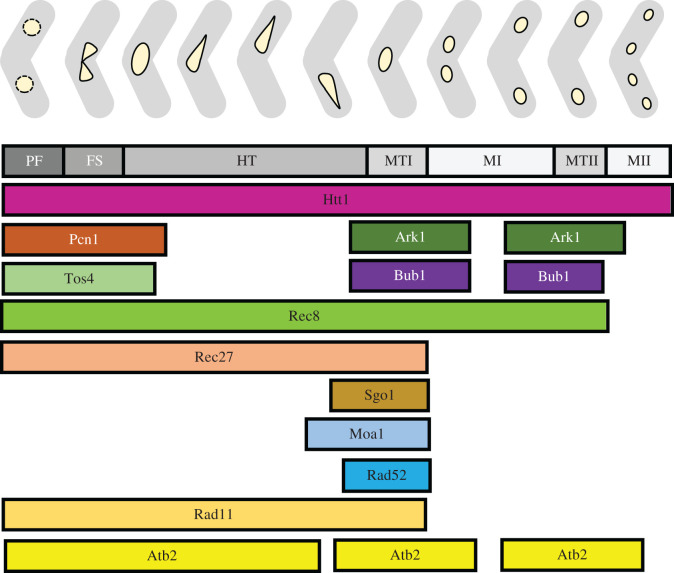
Meiotic signposts. Model presenting a visual atlas of reliable meiotic proteins that can be used to study the nuclear dynamics and disruptions of different meiotic processes in *S. pombe.* Meiotic signposts encompass events including PF, FS, HT, MTI, MI, MTII and MII. Respective timing of the presence of different meiotic proteins during described meiotic signposts is presented.

Fission yeast is an excellent model for meiotic progression. Typically, investigators describe the behaviour of one or a few novel proteins during meiosis, but protocols vary from laboratory to laboratory. In many cases, investigators make use of the *pat1-114* temperature sensitive mutant [15,93–95], which can induce a synchronous meiosis from haploid or diploid cells in response to temperature shift. However, *pat1*-driven meiosis has notable differences from a normal zygotic meiosis, and these are exacerbated in haploid meiosis compared to diploids (e.g. [14,16,96]). Additionally, many studies employ ‘snapshots’ of different timepoints, rather than continuous observations with live cell imaging that reveal dynamic and brief events. We have established a standardized protocol for imaging meiosis over time in normal homothallic or heterothallic strains, which we employed previously [17]. In this study, we chose representative proteins involved in different stages of meiosis, and compared their signal intensity, localization and dynamics against a standard panel of meiotic nuclear events including karyogamy, horsetail formation, MI and MII divisions. Based on these analyses, we present a reference that defines dynamic behaviour of proteins during meiotic DNA synthesis, genomic fusion, chromosome alignment, genetic recombination, metaphase and meiosis, using consistent conditions (figure 10). This live-cell microscopy approach provides information about the change of protein abundance and localization during meiotic differentiation and establishes a common template to facilitate comparisons of different proteins. This should prove particularly helpful in mutant backgrounds to define the extent of perturbations. By capturing the timing of different meiotic events in the context of nuclear change, we provide a general atlas of reliable markers that can be used to study dynamics and disruptions of meiotic processes in *S. pombe*.

## 4. Material and methods

### 4.1. Yeast strains, culture and cell growth

General fission yeast culture conditions, media compositions and strain constructions are used as described in [97]. Different fluorescently tagged *S. pombe* strains used in this study are listed in the electronic supplementary material, table S1. For live cell imaging, heterothallic (*h+* and *h−*) cells were initially grown in 5 ml YES media at 32°C for 12–16 h. 100–250 µl starter culture were transferred to 5 ml EMM media containing appropriate supplements and grown at 32°C until the culture reached a late log stage (1–1.2 × 10^7^ cells ml^−1^). Late log phase cells were harvested at 3000 r.p.m. for 5 min. To remove any remaining nutrient from the culture, pellets were washed twice with Malt Extract (ME) media. Finally, heterothallic pellets were resuspended in 10 ml ME media and incubated at 25°C for 12–16 h at 50–60 r.p.m. From this starved mating culture, 1 ml culture was harvested in microfuge tubes and used for live cell microscopy.

### 4.2. Fluorescence live-cell microscopy

Live cell imaging of meiotic events was performed as described in [17,19]. Briefly, 1 ml mating culture was harvested at 5000 rpm for 30 s. Pellets were resuspended in 250 µl ME media. 10 µl of cell suspension were spread on top of 2% agarose pad made with liquid sporulation media and sealed with VaLaP (Vaseline/ Lanolin/ Paraffin in a ratio 1 : 1 : 1 by weight). Live cell imaging was performed at 25°C (pre-calibrated chamber) on a Delta Vision Microscope (Applied Precision, GE Healthcare, Issaquah, WA) equipped with Olympus 60x/1.40 Plan-Apo objective lens, solid-state illuminator, and 12-bit Photo metrics CoolSNAP_HQ2 Charged-coupled device (CCD) camera. Different filter sets and exposure timings were used to excite and detect fluorescent proteins (supplementary table S1). 13 optical *z*-sections of 0.5 µm step size were acquired for each field at 10-minute intervals over 8 h. Images were acquired, deconvolved and all z-stacks projected into a single-plane as maximum intensity projection by SoftWoRx Version 5.5.1 (GE, Issaquah, WA) software. Finally, the projected fluorescence images were fused with transmitted light images. Downstream processing and image analysis were done using Fiji (ImageJ), an open source image analysis software [98]. Detailed description of image analysis is given in [17,19].

### 4.3. Statistical analysis

For different marker metrics across time and timing of individual events, the mean with 95% CI is presented. For comparisons among time points per marker, significance was evaluated using an ordinary One-Way ANOVA (for matched data) followed by Tukey's *post hoc* test. GraphPad Prism 8.4.2 was used for statistical analysis and graphical representations of the data.

## Data accessibility

Primary data, materials (such as statistical tools, protocols, software) and code will be publicly available either in the published manuscript or the electronic supplementary material.
